# Immune Checkpoint Inhibitors as Therapy to Down-Stage Hepatocellular Carcinoma Prior to Liver Transplantation

**DOI:** 10.3390/cancers14092056

**Published:** 2022-04-19

**Authors:** Nitin N. Katariya, Blanca C. Lizaola-Mayo, David M. Chascsa, Emmanouil Giorgakis, Bashar A. Aqel, Adyr A. Moss, Pedro Luiz Serrano Uson Junior, Mitesh J. Borad, Amit K. Mathur

**Affiliations:** 1 Department of Surgery, Division of Transplant and HPB Surgery, Mayo Clinic, Alix School of Medicine, Phoenix, AZ 85054, USA; moss.adyr@mayo.edu (A.A.M.); mathur.amit@mayo.edu (A.K.M.); 2 Department of Medicine, Division of Gastroenterology & Transplant Hepatology, Mayo Clinic, Alix School of Medicine, Phoenix, AZ 85054, USA; lizaola-mayo.blanca@mayo.edu (B.C.L.-M.); chascsa.david@mayo.edu (D.M.C.); aqel.bashar@mayo.edu (B.A.A.); 3 Department of Surgery, Division of Transplantation, University of Arkansas for Medical Sciences, Little Rock, AR 72205, USA; egiorgakis@uams.edu; 4 Center for Personalized Medicine, Hospital Israelita Albert Einstein, Sao Paulo 627, Brazil; pedroluiz_uson@hotmail.com; 5 Department of Medicine, Division of Hematology Oncology, Mayo Clinic, Alix School of Medicine, Phoenix, AZ 85054, USA; borad.mitesh@mayo.edu

**Keywords:** Hepatocellular Carcinoma (HCC), immune checkpoint inhibitors, nivolumab, neoadjuvant therapy, Barcelona Clinic Liver Classification (BCLC), liver transplant, cirrhosis

## Abstract

**Simple Summary:**

Hepatocellular Carcinoma (HCC) is one of the most common malignancies in the world with increasing prevalence. This review addresses the current and growing body of literature on the use of immune checkpoint inhibitors in patients with advanced HCC prior to liver transplantation and discusses many of the ongoing questions that must still be answered. Clearly there is a role for immunotherapy in HCC and further clinical trials will help guide the indications and parameters for their use.

**Abstract:**

Hepatocellular Carcinoma (HCC) is the most common liver malignancy and third leading cause of cancer death worldwide. For early- and intermediate-stage disease, liver-directed therapies for locoregional control, or down-staging prior to definitive surgical therapy with hepatic resection or liver transplantation, have been studied broadly, and are the mainstays of current treatment guidelines. As HCC incidence has continued to grow, and with more patients presenting with advanced disease, our current treatment modalities do not suffice, and better therapies are needed to improve disease-specific and overall survival. Until recently, sorafenib was the only systemic therapy utilized, and was associated with dismal results. The advent of immuno-oncology has been of significant interest, and has changed the paradigm of therapy for HCC. Lately, combination regimens including atezolizumab plus bevacizumab; durvalumab plus tremelimumab; and pembrolizumab plus Lenvatinib have shown impressive responses of between 25–35%; this is much higher than responses observed with single agents. Complete responses with checkpoint inhibitor therapy have been observed in advanced-stage HCC patients. These dramatic results have naturally led to several questions. Can or should checkpoint inhibitors, or other immunotherapy combinations, be used routinely before resection or transplant? Is there a synergistic effect of immunotherapy with locoregional therapy, and will pre-treatment increase disease-free survival after surgical intervention? Is it immunologically safe to use these therapies prior to transplantation? Much is still to be learned in terms of the dosing, timing, and overall utility of the use of immune checkpoint inhibitors for pre-transplant care and down-staging. More studies will be needed to understand the management of adverse events while maximizing the therapeutic window of these agents. In this review, we look at the current data on therapy with immune checkpoint inhibitors in advanced HCC, with a focus on pre-transplant treatment prior to liver transplant.

## 1. Introduction

Hepatocellular Carcinoma (HCC) is a serious complication of cirrhosis and the most common malignancy among primary liver cancers. According to the World Health Organization, HCC is the third leading cause of cancer-related death [1]. We have effective and well-established therapies for early- and intermediate-stage disease, including surgical resection, locoregional therapy with chemoembolization, yttrium (y-90) beads, ablation, and liver transplantation. Liver transplant remains a possible curative intervention. However, patients are typically excluded from transplants based on specific criteria, including tumor size/number and elevated alpha-fetoprotein. Immunotherapy has revolutionized the treatment of Hepatocellular Carcinoma, and may allow for tumor down-staging to within the transplantable criteria, even in advanced stage cases; however, the risk of severe allograft rejection and adverse events, and the potential for treatment-related death, have limited this possibility.

### Methods

Given the paucity of data on this subject, broad search criteria were used. MEDLINE, Google Scholar, Clinicaltrials.gov, and FDA.gov databases were queried to identify both published studies and assessments of ongoing trials. Search terms included: immunotherapy, immune checkpoint inhibitors, Hepatocellular Carcinoma, Barcelona Clinic Liver Classification (BCLC), and liver transplant, with inclusion and exclusion of neoadjuvant therapy. Results were filtered by English language, then assessed for relevance to this topic.

## 2. Background

Despite HCC being so common worldwide, there are variations in incidence and prevalence based on geographical area, with different inciting causes and treatment paradigms. HCC is the most common malignancy among primary liver cancers (75–95%) [2]. In the US, its incidence is expected to continue to increase through 2030, mainly in Hispanics, blacks, and whites [3,4]. Cirrhosis represents the strongest risk factor for the development of HCC [5]. The current guidelines recommend HCC screening every 6 months in patients with cirrhosis [6]. In North America and Europe, the primary drivers of HCC development are long-term hepatitis C infections, as well as the rise in obesity and diabetes, resulting in non-alcoholic steatohepatitis (NASH) and alcoholic liver disease. In much of Asia and Africa, hepatitis B is the most significant contributor to liver cancer. The BRIDGE (Bridge to Better Outcomes in HCC) study looked at the global patterns and variation in management of HCC and included data from 42 centers in 14 different countries [7]. The group alluded to the clear need for better surveillance to identify HCC earlier, as well as the necessity for the improved treatment of advanced disease. They noted that up to 70% of HCC presentation with advanced disease was limited to palliative treatment options, where the median overall survival for the Barcelona Clinic Liver Classification (BCLC) stages C and D was 15 and 4 months, respectively. In fact, per the BRIDGE Study, the most common BCLC stage at diagnosis was C in North America, Europe, China and South Korea, and A in Taiwan and Japan [7]. Despite the significant variety, locoregional therapy was the most frequent treatment across all BCLC stages around the world (likely related to later presentation); the exceptions were Taiwan, where resection was the first treatment, and Japan, where percutaneous ethanol injection or radiofrequency ablation were the preferred initial treatments. The BRIDGE group found transarterial chemoembolization (TACE) to be the most common type of locoregional therapy for BCLC stage C, and palliation for stage D [7]. For palliation, sorafenib was the proven systemic agent, with the noted benefit of increasing the median overall survival by only 2–3 months [8]. Despite the American Association for the Study of Liver Disease (AASLD)/European Association for the Study of the Liver (EASL) guidelines recommending sorafenib in BCLC Stage C disease, the BRIDGE study found low use worldwide in these patients, perhaps due to the low resources and toxicity profile, and the minimal benefit [9].

With the growing prevalence of HCC, advanced disease that is outside the standard treatment paradigm of liver resection or transplantation is increasingly common. Adjunct treatment with locoregional therapies has allowed tumor down-staging and future remnant growth, and has provided bridges to potential curative therapy in surgery. If fact, in some cases with less advanced disease, locoregional therapy can be curative [10,11].

## 3. Staging

Not only do patterns vary worldwide in HCC prevalence and treatment, but multiple staging systems are used to delineate disease burden. How do we actually define advanced HCC in a practical manner? The multiple staging systems include Okuda (1985) [12], the Barcelona Clinic Liver Cancer Classification system (BCLC, 1999) [13], Tumor-Node-Metastasis (TNM, 2002) [14], Tokyo (2005) [15], the Hong-Kong Liver Cancer classification (HKLC, 2014) [16], and others [17]. There have been several studies comparing these different classification systems, with numerous advantages and disadvantages noted for each system. According to the HCC practice guidance document from the AASLD/EASL, the BCLC classification system should be used. It incorporates not only tumor characteristics, but also the degree of liver dysfunction and performance status of the patient [9,18,19]. The initial BCLC system included the Child–Pugh score, the number and size of the nodules, evidence of vascular invasion or metastatic spread, and performance status, creating five prognostic subclasses from 0 (very early stage) to D (terminal stage) [13]. However, a recent study by Parikh et al. in 2018 compared several different staging systems, including the following systems: Italian Liver Cancer (ITA.LI.CA), HKLC, BCLC, Cancer of the Liver Italian Program (CLIP), and the model to estimate survival in ambulatory patients with Hepatocellular Carcinoma (MESIAH). They found that the HKLC and MESIAH systems outperformed BCLC in terms of discriminatory ability when looking at HCC data from the US retrospectively [20].

There are an overwhelming number of classification systems, each with its own strengths and weaknesses. For now, the BCLC criteria are the most widely used. In the future, perhaps with the addition of more biomarkers or tissue information, a better prognostic system will be possible.

**Figure 1:**
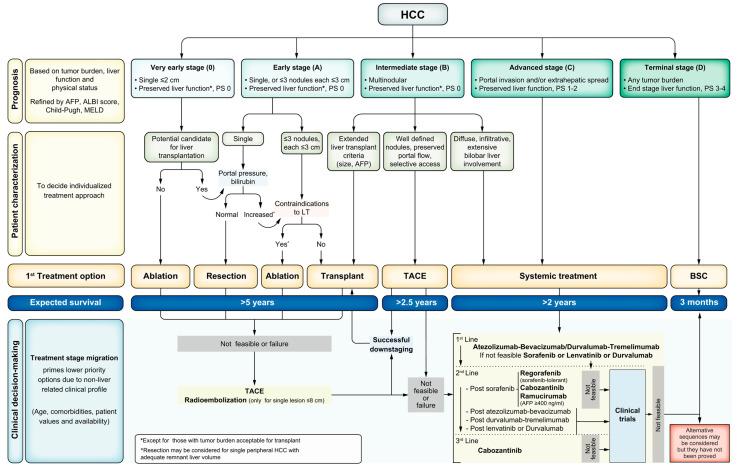
BCLC Staging (0-D) and Treatment Strategy 2022 Update.

For this review, we will consider BCLC stages B, C, and D as our advanced-to-end stages for HCC. The BCLC staging criteria have been updated along the way, with the most recent iteration released in 2022, where further factors have been used to group patients in the same stages 0-D (Figure 1). These include the use of the albumin–bilirubin score (ALBI) and alpha-fetoprotein (AFP). Stage B, an intermediate stage, is defined as multifocal HCC with preserved liver function, a performance status of 0 (no cancer-related symptoms), and no vascular invasion or distant spread. The magnitude of tumor burden is heterogenous in this group, which is broken into three sub-classes: a group that falls within the extended liver transplant criteria based on size/AFP; a group characterized by a well-defined tumor burden with preserved portal flow and targets for liver-directed therapy; and a class with diffuse infiltration that precludes effective locoregional therapy, thus making systemic therapy the only potential treatment option. BCLC C, an advanced stage, presents with vascular invasion or extrahepatic spread, and preserved liver function with a reasonable performance status of 0–2. These patients are directed toward systemic therapy. Finally, stage D includes patients with significant performance status impairment greater than 2 and/or significant liver dysfunction, without the option for liver transplant (either due to HCC burden or non-HCC factors). In these cases, palliative management with symptom control and comfort are the focus. Median survival data from the most up-to-date studies show the potential for more than 5 years for the transplantable stage B, greater than 2.5 years for the more advanced stage B, more than 2 years for stage C, and only 3 months for stage D [21].

Since there is wide variation in HCC clinical presentation within BCLC classes once granular clinical details are identified, it is necessary to tailor HCC management to each individual patient using a multidisciplinary tumor board, which is supported by the BCLC 2022 update [21]. When considering the question of where upfront therapy for liver transplantation would be most beneficial, the focus for the development of novel treatment protocols should be the selection of BCLC Stage B and C patients with varying presentations of tumor burden, prior local–regional therapy, possible resection, and potential previous systemic therapy.

## 4. Alpha-Fetoprotein

When it is expressed by HCC tumor cells, which is about 70% of the time [22], alpha-fetoprotein (AFP) is an excellent biomarker for monitoring HCC progression, recurrence, and response to therapy; it is an effective prognosticator of HCC behavior [23,24,25]. Its sensitivity and specificity in prognosticating disease progression has led it to be utilized in US liver allocation policy and in the assignment of waitlist priority. HCC patients in the US are given MELD exceptions to reflect their risk of mortality, which are linked to the AFP. Under the current US system, AFP greater than 1000 ng/mL portends a poor prognosis with a 50% recurrence rate, and patients are ineligible for the standardized MELD exception; AFP ≥ 500 ng/mL following locoregional treatment necessitates national liver review board referral, to determine MELD exception and point assignment; and AFP < 500 ng/mL is typically transplant-eligible or transplant-eligible following down-staging into Milan criteria with locoregional treatment [26]. Historically, the overall HCC recurrence rate in patients within Milan Criteria has been estimated to be 11–18% [27]. AFP greater than 50–100 ng/mL has been associated with higher rates of recurrence, as are other factors including explant pathology (notable for poorly differentiated tumors), a size ≥ 5 cm, and lymphovascular and capsular invasion [28,29].

## 5. Systemic Therapy

With the advent of immuno-oncology (IO), dramatic anti-tumor effects have been observed in a number of cancers including melanoma, non-small-cell lung cancer, and urothelial cancers related to the administration of this new class of immunotherapeutic drugs. Immunotherapy refers to multiple drug classes that upregulate one’s innate immune system to augment anti-tumor immunity and increase tumor cell death [30]. Until recently, the only proven systemic agent for hepatocellular cancer had been sorafenib [8,31]. In the past few years, similar agents including cabozantinib [32], lenvatinib [33], and regorafenib [34]—all multi-targeted tyrosine kinase inhibitors—have shown survival benefits. In addition, the vascular endothelial growth factor receptor-2 inhibitor, ramucirumab, has been felt to be efficacious and has survival benefits [35].

Immunotherapy agents can be classified into many categories. In advanced HCC, the immune modulation antibodies that block immune regulatory checkpoints and chimeric antigen receptor (CAR)-modified T cells are the most studied to date [36]. The first group are the anti-programmed cell death 1 (PD-1) and anti-programmed cell death ligand 1 (PD-L1) agents (Table 1). Nivolumab was one of the earliest studied and was first administered, in October of 2006, as an infusion to patients with advanced refractory malignancies including melanoma, renal cell carcinoma, and non-small-cell carcinoma [37]. These early experiences had limited toxicity and led to the eager study and use of pembrolizumab in April of 2011 for similar diseases [36]. Soon after, nivolumab was granted accelerated FDA approval as a second-line treatment for HCC [38]. Pembrolizumab as a single agent is currently FDA-approved for previously treated advanced HCC [39]. Finally, the combination of nivolumab with ipilimumab is another FDA-approved treatment for previously treated HCC [40]. However, on 29 April 2021, FDA’s Oncologic Drugs Advisory Committee voted to oppose maintaining the accelerated approval of nivolumab monotherapy for patients with advanced Hepatocellular Carcinoma who had received prior treatment with sorafenib, based on negative results [41]. Clearly, there is still much to be learned about these medications as single and combination agents in the advanced HCC space.

In terms of side-effect profiles and adverse events, multiple studies across several tumor types have led to some consensus guidelines in the management of these immune-related adverse events (IRAEs). Skin, gut, endocrine, lung, and musculoskeletal side effects are the most common, while cardiovascular, hematologic, renal, neurologic, and eye are much less frequent. Treatment-related death occurs in up to 2% of those being treated varying depending on the specific medication. Most of these effects have delayed onset and prolonged duration, even after drug cessation, but typically occur within 3–6 months of initiation [42]. Overall, IRAEs are frequent across multiple malignancies, with up to 70% [43,44] occurrence in those receiving a PD-1/PD-L1 antibody and, 90% [45] in those receiving an anti-CTLA4 antibody [46]. The Society for Immunotherapy of Cancer Toxicity Management working group has written a consensus document of the presentation and management of immune-related adverse events when using this family of drugs. They have noted some of the most common presentations, as well as some of the rarer significant events. Fatigue and infusion-related reactions are common with these drugs. Maculopapular rash and pruritus are seen in up to 40% of patients. Diarrhea is very common and must be distinguished from colitis. Combination therapy seems to have a higher incidence of colitis than monotherapy. Hepatitis is seen in some patients, especially with nivolumab and ipilimumab during concurrent administration, and presents up to 30% of the time. This must be considered carefully for HCC patients who may have diminished liver function. Thyroid dysfunction and hypopituitarism are the two most common endocrine adverse events. Pneumonitis is relatively infrequent, seen in less than 5% of patients. Multiple adverse events are common in patients receiving immunotherapy, and should be monitored closely. Care and experience are vital in using this class of drugs, and since some adverse events can occur late, even after termination of these drugs, diligence is key to the health of these patients outside their primary diagnosis of HCC [42].

Monoclonal antibodies used for immunotherapy maintain therapeutic concentrations for extended periods owing to their longer metabolic half-lives. The half-life of atezolizumab is 27 days [47], similar to other anti-PD1/PD-L1 drugs, including nivolumab (27 days) [48] and pembrolizumab (26 days) [49]. Given the importance of T cells in mediating transplant immunological responses against the donor allograft, it is critical to understand the pharmacodynamic effects of these drugs in the transplant setting. Little is known about this subject in clinical settings, but serious adverse events and anti-tumor effects related to IO treatment have been noted several months after dose administration. This suggests that the effects of the T cell activity related to these agents are more durable than predicted by half-life alone [50]. As an example, in the KEYNOTE-006 trial for melanoma, some patients treated with pembrolizumab who achieved a partial response (PR) converted to a complete response (CR) after stopping the drug [51,52]. Similarly, around 20% of patients treated with nivolumab in CHECKMATE-067 for advanced melanoma reached CR after 4 years of follow-up [53]. These results highlight that utilizing IO before transplantation should be evaluated with caution considering that the necessary time without treatment before transplantation is unknown. Studies evaluating drugs to oppose the IO effect are being developed. In a case report, abatacept, a cytotoxic T-lymphocyte-associated antigen 4 [CTLA-4] agonist, showed the capability to resolve a severe case of glucocorticoid-refractory myocarditis that was induced by the immune checkpoint inhibitor nivolumab [54].

## 6. HCC Clinical Trials

With the transition to immunotherapeutic agents in cancer treatment for multiple tumor types, a plethora of clinical immunotherapy trials have been completed, with promising results. More recently, advanced HCC has been studied; there are many ongoing trials using sorafenib as a systemic benchmark for the standard of care, with some key results (Table 2). Chronologically, one of the first IO clinical trials for advanced liver cancer was the CHECKMATE-040 trial, which began accrual in 2012, and initially evaluated nivolumab versus sorafenib in advanced liver cancer. It is an active but not-recruiting study looking at further comparisons of nivolumab in combination with other agents for advanced HCC. Data from this study were first published in 2017 in a dose-escalation and -expansion study. Nivolumab had a reasonable safety profile with 15–20% response rates in the dose expansion/escalation phases, even with three complete responses. This study phase only included cirrhotic patients with no signs of hepatic decompensation [38]. More recent data from this trial showed similar safety in Child’s B patients as in a Child’s A setting using nivolumab. Some stabilization of liver function in Child’s B patients, and longer median survival compared to sorafenib at 7.6 vs. 2.5–5.4 months, were observed [55]. The combination therapy with ipilimumab was evaluated in a cohort from this trial as well. An arm A regimen (4 doses 1 mg/kg nivolumab plus 3 mg/kg ipilimumab every 3 weeks, then 240 mg nivolumab every 2 weeks) reached a response rate of 32% [40]. CHECKMATE-459 evaluated nivolumab versus sorafenib as a first-line agent for advanced HCC. The study did not find a statistically significant difference in overall survival between the two drugs, but there was good clinical activity of the drug and a reasonable safety profile. Their conclusion was that nivolumab could be a good first-line agent instead of sorafenib [56].

The KEYNOTE trials all looked at the use of pembrolizumab in advanced HCC. KEYNOTE-224 was an international non-randomized study that evaluated pembrolizumab’s use in patients with preserved liver function who were already treated with sorafenib as a first-line therapy, but had progression of HCC disease or were intolerant to therapy. They found an objective response in 17% of patients and stability in an additional 44%. There were adverse events noted in 73% of patients, but they were mostly grade 1–2 in severity. The study group concluded that that pembrolizumab is well tolerated and could be considered as an effective treatment agent in advanced HCC [39]. KEYNOTE-240 randomized patients to take pembrolizumab versus a placebo, to study overall and progression-free survival of those who had previously been treated with sorafenib. Though the data did not reach statistical significance, they found a trend of overall survival at 13.9 months versus 10.6 months for the placebo, and felt that the trial demonstrated a favorable risk-to-benefit profile.

As of late, sorafenib has been surpassed in efficacy in front-line therapy in randomized phase III trials using immunotherapy combination regimens. Combinations of anti-PD1/PD-L1 antibodies with antiangiogenics or anti-CTLA4 have been proven to be highly effective. The HIMALAYA study evaluated the combination of durvalumab and tremelimumab as a first-line treatment in patients with advanced HCC. The data presented confirmed the superiority of the regimen compared to sorafenib, plus the non-inferiority of a durvalumab single agent against sorafenib [63]. Most recently, data were presented at the ASCO GI meeting in 2022 [63]. In the trial, 393 patients with advanced or unresectable HCC were treated with durvalumab plus a single-dose tremelimumab, and 389 were treated with sorafenib. At a median follow-up of 33 months, the median OS was statistically improved with the IO combination, as was the median OS at 16.4 months with the combination, versus 13.8 months with sorafenib (HR 0.78, *p* = 0.0035). The objective RR was 20% with the combination, and 5% with sorafenib. Furthermore, the study was powered to evaluate the noninferiority of the durvalumab single agent against sorafenib. The objective was achieved, and the median OS of durvalumab was 16.6 months against 13.8 months with sorafenib (HR 0.86 (0.73–1.03), where the noninferiority margin was 1.08.

The IMBRAVE150 trial evaluated the combination of the anti-PD-L1 atezolizumab with the anti-VEGF bevacizumab against sorafenib for advanced HCC [60]. In the trial, 336 patients were treated with the combination, and 165 patients were treated with sorafenib. The overall survival (OS) at 12 months was 67.2% with atezolizumab/bevacizumab and 54.6% with sorafenib. The median progression-free survival (PFS) was 6.8 months and 4.3 months in the respective groups (HR 0.59; 95% CI, 0.47–0.76; *p* < 0.001). The median OS was not reached in the patients who received atezolizumab plus bevacizumab, and was 13.2 months in the sorafenib group (HR 0.58; 95% CI: 0.42–0.79; *p* = 0.0006). An extended follow-up date showed statistically significant overall survival at 19.2 months for atezolizumab with bevacizumab versus 13.4 months for sorafenib [61]. The overall response rate with the combination was 33%. Based on these results, on 29 May 2020, the FDA approved the atezolizumab/bevacizumab combination for patients with unresectable or metastatic Hepatocellular Carcinoma who have not received prior systemic therapy.

Finally, LEAP-002 evaluated the combination of pembrolizumab and lenvatinib as a first-line treatment for those with advanced HCC. Recent data in the phase Ib portion of the study noted an objective response rate of 36% in patients, and a disease control rate of 88% in 100 treated patients. This combination appears to be highly effective in advanced unresectable HCC [64].

These trials focused on advanced or unresectable HCC (BCLC C and D) and demonstrated the safety and efficacy profiles of these medications in patients with somewhat compensated liver disease without the intention for transplant [65]. The most recent BCLC guidelines suggest atezolizumab plus bevacizumab or durvalumab plus tremelimumab as first-line systemic therapies [21].

Two newer trials, PLENTY202001 and DULECT2020-1, both based in China, are looking at combination therapy for upfront use prior to liver transplantation; it will be important to follow these as we look to define treatment regimens before transplant. Important considerations include dosing, the combination of therapies with locoregional approaches, addressing liver decompensation, and the timing of immunotherapy cessation before consideration. Pembrolizumab and Lenvatinib in Participants with Hepatocellular Carcinoma (HCC) Before Liver Transplant (PLENTY202001) is an unmasked randomized controlled trial (n = 192), evaluating the safety and efficacy of pembrolizumab in combination with lenvatinib as an upfront therapy for HCC patients beyond the Milan criteria awaiting a transplant. The hypothesis of this trial is that neoadjuvant pembrolizumab plus lenvatinib is superior to the standard-of-care waitlisting practice with regard to the recurrence-free survival and objective response rate. The treatment arm is to receive pembrolizumab in intravenous cycles such that it is stopped 42 days or more before transplant, and to receive Lenvatinib orally with cessation at least 7 days before transplant [66]. Durvalumab and Lenvatinib in Participants with Locally Advanced and Metastatic Hepatocellular Carcinoma (Dulect2020-1) is a prospective open-label trial studying 20 patients with locally advanced HCC before liver transplant or metastatic HCC, looking at progression-free and recurrence-free survival. Of course, the limitations of any pre-transplant effort are the inability to control the timing of the transplant to aid with medication cessation, as well as justifying the use of organs in patients who do not down-stage appropriately in response to the IO. Time will tell if these patients were selected appropriately for transplant and if the combination therapies truly affected the outcome.

## 7. Case Reports

There are several case reports in the literature addressing the use of immunotherapy as a neoadjuvant treatment before liver transplantation (Table 3). Even though the use of PD-L1 inhibitors on advanced HCC has been rapidly increasing, their use in liver transplant candidates is conventionally discouraged due to reports of severe allograft rejection, graft loss, and even death [67,68,69]. Tabrizian et al. describe a single-center series of nine HCC patients who were successfully transplanted after receiving nivolumab as a neoadjuvant treatment. Most of their patients had liver resections as primary treatment, and were treated with nivolumab for recurrence. One-third of the patients exceeded the Milan criteria at some point in the pre-transplant course. These patients received steroids for induction and standard maintenance therapy, including a steroid taper, mycophenolate mofetil, and tacrolimus with a goal trough of 10–12 ng/mL. In one-third of the cases, the explant pathology showed a near-complete tumor response. Despite the fact that eight patients (89%) had their last nivolumab dose just 4 weeks prior to transplant, there were no documented episodes of severe allograft rejection, primary non-function of the allograft, graft loss, tumor recurrence, or death [70].

Schwacha-Eipper et al. report a patient with a 6.4 cm HCC and compensated cirrhosis, who underwent a laparoscopic liver resection with pathology revealing poorly differentiated HCC. Due to HCC recurrence, the patient was started on systemic therapy, but progressed to sorafenib and was intolerant to regorafenib. He completed 34 cycles of Nivolumab. The patient underwent a transplant 15 weeks after immunotherapy cessation without complications. The pathology showed a single, viable 4.2 cm poorly differentiated lesion. At one-year post-transplant, the patient had no tumor recurrence and no evidence of allograft rejection [71].

Our transplant group at the Mayo Clinic in Arizona reported on the utilization of immunotherapy as a “neoadjuvant” therapy prior to liver transplant. The patient had well-compensated alcohol-related cirrhosis, but developed two HCC lesions within the Milan criteria. Despite Y-90 radioembolization, his AFP rose to greater than 1000 ng/mL and new HCC lesions developed, precluding transplant candidacy. Sorafenib was ineffective, and the patient was transitioned to nivolumab with ipilimumab. After six months of immunotherapy, his AFP fell from over 10,000 ng/mL to 19.6 ng/mL, with no new HCC lesions. He developed hepatic decompensation and, given his response to therapy, was listed for transplant with a native MELD-Na of 30. As severe allograft rejection has been reported. The immunotherapy was stopped before transplant to reduce the risks [74]. A successful, rejection-free transplant occurred 9 weeks later. The pathology revealed no evidence of residual HCC, and the patient has remained cancer free with no evidence of rejection at 12 months [72].

The most recent reported series is from the transplant team at the University of California, San Diego (UCSD), and references five cases with the use of nivolumab in patients with T2 and T3 tumors. The range of withdrawal was from ten days to six months, and they used rabbit anti-thymocyte globulin in three cases for induction immunosuppression, and in one case post-transplant to salvage the allograft secondary to rejection. They found success with use of the nivolumab, and four of the five patients are currently alive, with one requiring retransplant secondary to massive hepatic necrosis. UCSD’s conclusion was that the withdrawal of nivolumab at three months or more did not seem to increase the incidence of rejection in post-transplant patients, and that is could be used successfully in a neoadjuvant setting [73].

These clinical experiences in real-world practice suggest a role for immunotherapy in a pre-transplant setting for HCC treatment. In each of these reported cases, nivolumab was the mainstay of immunotherapy, and most groups employed an immunotherapy stoppage several weeks in advance of transplant to mitigate the risk of profound rejection by activated host T-cells.

## 8. Discussion

With the increased prevalence of intermediate and advanced HCC, further additions to the treatment paradigm are needed. The advent of the immunotherapy era has significant promise for reducing the burden of HCC-related morbidity and mortality in those with significant tumor burden. For selected patients with cirrhosis and early-stage HCC, liver transplantation is an excellent therapeutic option associated with low recurrence rates and excellent survival. The use of locoregional therapy for HCC prior to liver transplant is associated with lower rates of waitlist dropout due to excellent local control, and has allowed the down-staging of patients with intermediate and advanced HCC into Milan criteria with excellent results. The success of “down-staging” with locoregional therapy and the impressive results of immunotherapy trials in advanced HCC has naturally encouraged HCC care-providers to ponder whether immunotherapy can be applied as a down-staging therapy. Indeed, intrepid transplant and HCC tumor programs have embarked on off-label use of immunotherapy as “accidental neoadjuvant” therapy, where the indication is advanced disease and immunotherapy is used as the destination therapy. The transition to the transplant pathway is applied only after dramatic clinical responses have occurred. Based on these anecdotal reports, it is far from obvious how immunotherapy will fit in as a neoadjuvant therapy prior to transplant, whether it will be used in combination with other treatment modalities, and what will emerge as the most effective immunotherapeutic strategies for first- and second-line therapy. Despite this, recent UNOS policy updates have considered these data and have concluded that immunotherapy use should not preclude the consideration for HCC exception points in the US [75]. Current data indicate that combination therapy with atezolizumab plus bevacizumab or durvalumab plus tremelimumab are efficacious and should be considered as first-line systemic therapy. As further clinical trials are completed, we will likely see which single agent or combinations of immunotherapy will be the first- and second-line treatments for higher-risk HCC Cases. Safety profiles will emerge as these agents are applied in patients with varying degrees of liver dysfunction.

Importantly, more knowledge of immunotherapy outcomes in advanced HCC will pave the way for prospective clinical trials and protocols where these agents may be applied intentionally, a priori, as a bridge to liver transplantation. Trials are needed to better understand whether liver transplant with immunotherapy provides a benefit above immunotherapy alone or transplant alone. Further, when considering neoadjuvant immunotherapy, more information on the selection of patients and delineation of higher-risk cases early in the disease timeline will be critical; how to select the right patients for systemic therapy and when to start and end are still to be defined. At present, there are many more questions than answers. For clinicians, the multiple HCC staging classifications confuse the best treatment approach. BCLC classification provides guidance and has been the standard for systemic therapy trials, but will newer HCC staging classifications such as those using biomarkers, tissue and liquid biopsies be better in selecting patients for immunotherapy and, subsequently, for transplant? Multiple systemic immunotherapy agents may be effective in advanced HCC patients with the most aggressive HCC tumor biology—for example, those who present beyond Milan criteria, those with recurrence or new lesions after resection, and those with disease progression despite locoregional liver-directed therapies. What will serve as the standard to direct patients toward transplant? Will time with stable disease on immunotherapy be the most predictive factor of good post-transplant outcomes, will it be the length of overall treatment, or will a combination of locoregional therapy with systemic treatment be the golden ticket? We need to understand tumor burden, vascular involvement, the degree of intrinsic liver disease, and the functional status of the patient to assess the prognosis and likelihood of tolerating and benefiting from different treatments. Trials should implement and use biomarkers such as AFP, AFP-L3, and DCP. However, future trials may employ a more nuanced approach, capturing tumor biology with needle or liquid biopsies as we learn more about their utility as intermediate trial endpoints or secondary outcomes. Significant effort in developing such trials is warranted, particularly in contexts where both deceased- and living-donor liver transplantation is available.

An additional clinical question is whether immunotherapy should be used in the neoadjuvant setting alone or in combination with local control strategies prior to transplant. This is answerable through clinical trials and rigorous multi-center prospective studies. Local control strategies, including resection or other liver-directed therapies such as embolization, radioembolization, or ablation, have been widely utilized in early-stage disease to reduce the risk of recurrence. Their inherent technical success and durable response has led them to be utilized in intermediate-stage and advanced HCC by BCLC staging. For patients waitlisted for transplant for HCC, since Mazzaferro’s seminal paper in 1996, the Milan Criteria (single HCC ≤ 5 cm or up-to-3 HCCs ≤ 3 cm, without vascular invasion) have been widely recognized as the benchmark for selecting cirrhotic patients for orthotopic liver transplantation for the treatment of HCC [76]. However, patients have been successfully down-staged using local control strategies and achieved excellent outcomes with transplant. A large analysis—based on the Scientific Registry of Transplant Recipients—on the post-LT outcomes of HCC patients classed as beyond Milan vs. within Milan vs. all-comers, indicated a higher risk of recurrence in the latter group (6.9% vs. 12.8% vs. 16.7%, respectively); however, a 71.4% 3-year survival rate was achieved in the all-comers group. A recent multicenter retrospective study on HCC down-staging reported an overall survival and recurrence rate in down-staged patients after LT of 64.3% vs. 71.3%, and 18.7% vs. 11.1%, compared to patients within the Milan Criteria, respectively [77]. These are just a small fraction of the studies that have been written on the value of locoregional therapy in early and intermediate stage HCC prior to liver transplant. In this context, the use of locoregional therapy for HCC in the liver transplant paradigm is deeply imprinted in the transplant community. In the studies reported here on the utilization of “neoadjuvant” nivolumab, locoregional therapies were used. The true clinical value of neoadjuvant locoregional therapy in combination with neoadjuvant immunotherapy prior to liver transplant remains an open question, and one that should be studied rigorously in clinical trials.

When considering immunotherapy prior to transplant, an additional area of inquiry is to determine whether immunotherapy will predictably impact graft function after transplant, and how providers should approach the timing of surgery and post-transplant medication management. The use of immunotherapy may create challenges for induction and maintenance immunosuppression related to the transplant. The case reports noted in this article emphasize the need for immunotherapy cessation prior to transplant, to pre-empt activated host T-cell responses against the allograft. Important areas for future studies to address are the risks and benefits of anti-thymocyte therapies for induction in this setting. Even in the absence of immunotherapy, T-cell-depleting agents are associated with increased risk of opportunistic infections and post-transplant lymphoproliferative disorders. In contrast, their efficacy may help facilitate deceased-donor liver transplant. Deceased-donor liver transplantation has an uncertain time horizon, which complicates the decision of when immunotherapy should be stopped pre-transplant. The use of T-cell-depleting agents may provide some protection from anti-allograft responses in the setting of ongoing drug activity. Studies should also be deliberate in maintenance-immunosuppression regimens with steroids, mycophenolate, and calcineurin inhibitors, to ensure that both the trial end-points related to recurrence and adverse event risks in the trial arms remain unbiased.

## 9. Conclusions

With the increasing prevalence of Hepatocellular Carcinoma worldwide and significant cancer-specific mortality, we need improvements in intermediate and advanced HCC management. An important potential therapeutic approach is to couple immunotherapy with bridge-to-transplant to prolong survival, but this approach needs to be tested against immunotherapy alone, as well as transplant alone. With regard to systemic therapy, sorafenib has been the mainstay, but recent trials have shifted toward immunotherapy for advanced disease. Given the dramatic responses that have been observed, immunotherapy may also serve in the neoadjuvant realms prior to liver transplant and the adjuvant, or in the palliative realm. Multiple studies are currently being performed with single immunotherapy agents and combinations of them, and some promising outcomes have been reported with neoadjuvant immunotherapy and transplant. However, some caution is warranted to allow for more clinical trials that will help address important questions. These questions include efficacy based on randomized trial models; if efficacious, more detailed questions are warranted regarding the best timing of immunotherapy cessation prior to transplant, and how transplant recipients should be managed with regard to induction and maintenance immunosuppression in these cases. We are on an excellent path forward; however, more studies are warranted to provide transplant and oncology providers with a holistic understanding of the benefits and risks of a neoadjuvant immunotherapy approach prior to liver transplant, as well as the potential for synergy with our current treatment strategies directed toward HCC.

## Figures and Tables

**Table 1:** Immune checkpoint inhibitors.

Class	Name	FDA Approval for HCC	FDA HCC Indication
PD-1	Nivolumab	Yes 22 September 2017	HCC previously treated with sorafenib
	Pembrolizumab	Yes 9 November 2018	HCC previously treated with sorafenib
	Sintilimab	No	NA
	Camrelizumab	No	NA
PD-L1	Atezolizumab	No	NA
	Avelumab	No	NA
	Durvalumab	No	NA
CTLA-4	Ipilimumab	No	NA
	Tremelimumab	No	NA
Combo	Nivolumab + Ipilimumab	Yes 10 March 2020	HCC previously treated with sorafenib
	Atezolizumab + Bevacizumab	Yes 29 May 2020	Unresectable or metastatic HCC
	Durvalumab + Tremelimumab	No	NA

Data from FDA.gov.

**Table 2:** Completed/ongoing relevant immune checkpoint inhibitor studies for advanced HCC.

Study	Title	Start Date	Drug Arms	Status	Completion Date
CHECKMATE-040 NCT01658878 [38,40,55,57]	An Immunotherapy Study to Evaluate the Effectiveness, Safety and Tolerability of Nivolumab or Nivolumab in Combination with Other Agents in Patients with Advanced Liver Cancer	October 2012	Nivolumab Sorafenib Nivolumab + Ipilimumab Nivolumab + Cabozantinib Nivolumab + Ipilimumab + Cabozantinib	Active Not Recruiting	December 2024
CHECKMATE-459 NCT02576509 [56]	An Investigational Immunotherapy Study of Nivolumab Compared to Sorafenib as a First Treatment in Patients with Advanced HCC	December 2015	Nivolumab Sorafenib	Active Not Recruiting	June 2022
NCT02519348 [58]	A Study of Durvalumab or Tremelimumab Monotherapy, or Durvalumab in Combination with Tremelimumab or Bevacizumab in Advanced HCC	October 2015	Durvalumab Tremelimumab Durvalumab + Tremelimumab Durvalumab + Bevacizumab	Active Not Recruiting	December 2022
KEYNOTE-224 NCT02702414 [39]	Study of Pembrolizumab as Monotherapy in Participants with Advanced HCC	May 2016	Pembrolizumab	Active Not Recruiting	June 2022
KEYNOTE-240 NCT02702401 [59]	Study of Pembrolizumab vs. Best Supportive Care in Participants with Previously Systemically Treated Advanced HCC	May 2016	Pembrolizumab Placebo	Completed	September 2021
KEYNOTE-394 NCT03062358	Study of Pembrolizumab or Placebo Given with Best Supportive Care in Asian Participants with Previously Treated Advanced HCC	April 2017	Pembrolizumab Placebo	Active Not Recruiting	December 2022
HIMALAYA NCT03298451	Study of Durvalumab and Tremelimumab as First-line Treatment in Patients with Advanced HCC	October 2017	Durvalumab Durvalumab + Tremelimumab Sorafenib	Recruiting	August 2024
IMbrave-150 NCT03434379 [60,61,62]	A Study of Atezolizumab in Combination with Bevacizumab Compared with Sorafenib in Patients with Untreated Locally Advanced or Metastatic HCC	March 2018	Atezolizumab + Bevacizumab Sorafenib	Active Not Recruiting	June 2022
NCT03755739	Trans-Artery/Intra-Tumor Infusion of Checkpoint Inhibitors for Immunotherapy of Advanced Solid Tumors (including HCC)	November 2018	Pembrolizumab Atezolizumab Ipilimumab Pembrolizumab + Ipilimumab Atezolizumab + Ipilimumab	Recruiting	November 2033
COSMIC-312 NCT03755791	Study of Cabozantinib in Combination with Atezolizumab Versus Sorafenib in Subjects with Advanced HCC Who Have Not Received Previous Systemic Anticancer Therapy	December 2018	Cabozantinib + Atezolizumab Sorafenib	Recruiting	December 2021
LEAP-002 NCT03713593	Safety and Efficacy of Lenvatinib in Combination with Pembrolizumab vs. Lenvatinib as First-line Therapy in Participants with Advanced HCC	December 2018	Pembrolizumab + Lenvatinib Lenvatinib + Placebo	Active Not Recruiting	December 2023
ORIENT-32 NCT03794440	A Study to Evaluate the Efficacy and Safety of Sintilimab in Combination with IBI305 (Anti-VEGF Monoclonal Antibody) Compared to Sorafenib as the First-Line Treatment for Advanced HCC	February 2019	Sintilimab + IBI305 Sorafenib	Active Not Recruiting	December 2022
EMERALD-2 NCT03847428	Assess Efficacy and Safety of Durvalumab Alone or Combined with Bevacizumab in High Risk of Recurrence HCC Patients After Curative Treatment	April 2019	Durvalumab Durvalumab + Bevacizumab Placebo	Recruiting	May 2024
NCT03764293	A Study to Evaluate Camrelizumab in Combination with Apatinib as First-Line Therapy in Patients with Advanced HCC	June 2019	Camrelizumab + Apatinib Sorafenib	Recruiting	June 2022
PLENTY202001 NCT04425226	Pembrolizumab and Lenvatinib in Participants with HCC Before Liver Transplant	August 2020	Pembrolizumab + Lenvatinib No systemic therapy	Recruiting	December 2024
DULECT2020-1 NCT04443322	Durvalumab and Lenvatinib in Participants with Locally Advanced (before liver tx) and Metastatic HCC	September 2020	Durvalumab + Lenvatinib None	Recruiting	December 2025
REACH-2 NCT02435433 [35]	A Study of Ramucirumab (VEGFR2 Inhibitor) Versus Placebo in Participants with HCC and Elevated Baseline Alpha-Fetoprotein	July 2015	Ramucirumab Placebo	Active Not Recruiting	December 2021

Additional Data from clinicaltrials.gov.

**Table 3:** Case Reports of Neoadjuvant Immunotherapy Use.

Group	Drug & Treatment Length	Withdrawal	Outcome
Mount Sinai Medical Center, Recanati/Miller Transplantation Institute, New York, New York [70]	There were 9 patients; 5/9 had prior resection and 3/9 were outside Milan Criteria.	8/9 had Nivolumab within 4 weeks of transplant	Bile leak in 1 and rejection in another attributed to low tacrolimus; explants > 90% tumor necrosis in 3/9 cases.
Department of Surgery, Division of Hepatobiliary Surgery & Liver Transplantation, Vanderbilt University Medical Center, Nashville, Tennessee [69]	Lap Resection; new disease within the liver revealed; started on Sorafenib and received y-90, then referred with rising AFP; received Nivolumab and TACE with afp down to 5.5 ng/mL with response. Remained within Milan × 1 year and was activated.	Nivolumab last dose 8 days before transplant	Fatal Hepatic Necrosis; death at POD #10; path showed no viable tumor on explant.
University Clinic for Visceral Surgery and Medicine, Inselspital Bern, Switzerland [71]	Lap resection, then sorafenib for 14 months, then REACH—II in placebo × 2 months; regorafenib × 11 weeks, then nivolumab and Ablation × 34 cycles.	Nivolumab stopped 6 weeks before activation	1 year post-OLT showing no evidence of recurrence; explant with viable 4.2 cm; poorly differentiated HCC.
Mayo Clinic Arizona Transplant Center, Phoenix, AZ [72]	Initial presentation of ETOH-related cirrhosis with 2 lesions within Milan; despite y-90 treatment AFP was 1164 to 3000 and was started on sorafenib; felt not to be a transplant candidate. AFP rose to >10,000 and was switched to Nivolumab + Ipilimumab with drastic response at 6 months, then transplanted.	Nivolumab + Ipilimumab stopped 8 weeks before listing	Received IV Steroids + thymoglobulin; path without any viable tumor; no rejection.
Division of Transplant and Hepatobiliary Surgery, Department of Surgery, University of California San Diego, San Diego California [73]	5 patients all given nivolumab prior to liver transplant with T2 or T3 HCC tumors; 3 patients received rATG for induction; 1 patient received rATG for salvage attempt after rejection.	Nivolumab withdrawn 10 days to 6 months before transplant	Total of 4/5 patients alive with one patient requiring retransplant secondary to massive hepatic necrosis; patients with withdrawal 3 months or greater had no evidence of rejection

rATG—rabbit anti-thymocyte globulin.

## Data Availability

Not Applicable.
